# Circular Dichroism and Fluorescence Spectroscopy of Cysteinyl-tRNA Synthetase from *Halobacterium salinarum* ssp. NRC-1 Demonstrates that Group I Cations Are Particularly Effective in Providing Structure and Stability to This Halophilic Protein

**DOI:** 10.1371/journal.pone.0089452

**Published:** 2014-03-03

**Authors:** Christopher J. Reed, Sarah Bushnell, Caryn Evilia

**Affiliations:** Department of Chemistry, Idaho State University, Pocatello, Idaho, United States of America; Russian Academy of Sciences, Institute for Biological Instrumentation, Russian Federation

## Abstract

Proteins from extremophiles have the ability to fold and remain stable in their extreme environment. Here, we investigate the presence of this effect in the cysteinyl-tRNA synthetase from *Halobacterium salinarum* ssp. NRC-1 (NRC-1), which was used as a model halophilic protein. The effects of salt on the structure and stability of NRC-1 and of *E. coli* CysRS were investigated through far-UV circular dichroism (CD) spectroscopy, fluorescence spectroscopy, and thermal denaturation melts. The CD of NRC-1 CysRS was examined in different group I and group II chloride salts to examine the effects of the metal ions. Potassium was observed to have the strongest effect on NRC-1 CysRS structure, with the other group I salts having reduced strength. The group II salts had little effect on the protein. This suggests that the halophilic adaptations in this protein are mediated by potassium. CD and fluorescence spectra showed structural changes taking place in NRC-1 CysRS over the concentration range of 0–3 M KCl, while the structure of *E. coli* CysRS was relatively unaffected. Salt was also shown to increase the thermal stability of NRC-1 CysRS since the melt temperature of the CysRS from NRC-1 was increased in the presence of high salt, whereas the *E. coli* enzyme showed a decrease. By characterizing these interactions, this study not only explains the stability of halophilic proteins in extremes of salt, but also helps us to understand why and how group I salts stabilize proteins in general.

## Introduction

Understanding the effects that environmental factors have on a protein's structure is a difficult task. A protein's structure, or fold, is influenced by many interactions. The most influential is the interaction of nonpolar residues that form the hydrophobic core of the protein. Electrostatic interactions between the protein's polar residues are able to form salt bridges and hydrogen bonds that essentially lock the protein into a particular configuration. While some proteins can spontaneously fold into the correct structure, others need external cues in order to fold correctly. In the cell, chaperonins play a major role in protein folding and structure [1]–[4]. They have the ability to change the protein's local environment and therefore change the way the protein folds. However, not all proteins will or can be folded by chaperonins. For example, extremophiles, organisms that thrive in extreme environments, can often utilize their environmental conditions to help fold their proteins [5]–[10]. Thermophiles, whose proteins have more hydrophobic residues and increased ionic interactions over non-thermophilic proteins, take advantage of the more favorable salt bridge interactions possible due to higher temperatures in order to fold their proteins [11]–[13]. Higher temperatures are a stabilizing force for thermophilic proteins and encourages folding with very slow unfolding pathways [12].

Halophiles, organisms that thrive in extreme amounts of salts, can also use the surrounding environment to their advantage. Electrostatic and hydrophobic interactions of a protein are changed when it is subjected to an extremely ionic environment. Electrostatic interactions, especially salt bridging, become unfavorable, due to their disruption by ions, while the hydrophobic effect between nonpolar residues is strengthened [14], [15]. It is not well understood how halophilic proteins remain stable in this kind of environment, though a common observation made about halophilic proteins is the large amount of acidic residues on the protein surface [14]–[16]. While the role these acidic residues have in adaptation is not well understood, it is thought to improve the protein's solubility in high concentrations of salt [13]. Another common halophilic adaptation is decreased protein hydrophobicity, including a reduction in the number and size of hydrophobic residues; this prevents the proteins from aggregating due to the increased hydrophobic effect in high salt conditions [13], [17], [18].

How these adaptations relate to the stability of halophilic protein structure is unknown. Previous research has demonstrated the importance of salt for halophilic protein folding [19]–[21]. An esterase in *Haloarcula marismortui* was found to have no secondary structure without salt, and to gain most of it in 2 M KCl. For this enzyme, salt-dependent folding was observed around 0.25 M KCl, where there was an increase in α-helical content [19]. Similarly, far-UV CD spectroscopy of RNase H1 from *Halobacterium salinarum* ssp. NRC-1 revealed partial secondary structure in no salt, but the amount of secondary structure increased in higher concentrations of NaCl, MgCl_2_, and MnCl_2_ [20]. The overall thermal stability of this RNase H1 also increased significantly in 3 M NaCl and a further increase was observed in 3 M NaCl and 300 mM MgCl_2_ [20]. This suggested that different salts have different effects on halophilic protein stability.

Because we are interested in how proteins maintain their structure and stability under molar concentrations of salts, a protein was chosen that contains known halophilic adaptions while still remaining similar to a non-halophilic homologue. For this reason, we chose to study the salt-dependent stability of the cysteinyl-tRNA synthetase from *Halobacterium salinarum* ssp. NRC-1. This enzyme catalyzes the attachment of cysteine to its cognate tRNA to generate a substrate for the ribosome. Because this is a required reaction in every cell, CysRS is highly conserved and, therefore, any evolutionary adaptations that the *H. salinarium* ssp. NRC-1 required for activity and stability in high salt should be more readily discernible. While adaptations to environmental extremes are not always obvious, a number of adaptations in NRC-1 CysRS to salt have been observed and characterized through sequence alignments with *E. coli* CysRS and homology modeling [6], [13], [22]. NRC-1 CysRS contains a significant amount of negative surface charge from the large number of aspartic and glutamic acid residues, similar to most other halophilic proteins. It also has a 19-residue insertion which is thought to give the enzyme flexibility in high salt, particularly near the active site [6]. The presence of peptide insertions has been seen in other halophilic proteins [15], [23], [24].

From past work, it was determined that NRC-1 CysRS loses half of its activity at KCl concentrations below 2 M [22]. While there is data to support a structural transition in the enzyme when the salt concentration varies, it was unclear if this was a structural effect or a change in the enzyme flexibility and/or stability [6]. Another question that arose was whether cations from group I and II had different effects on the structure or stability of halophilic proteins. In the literature, there is no clear answer as to whether one group I salt is better than another. Most research on halophilic proteins has suggested that there is no significant difference between sodium and potassium, even though halophiles tend to have a significantly higher intracellular concentration of potassium [13], [25]–[27].

In this work, we describe the effects of salts on the structure and stability of NRC-1 using far-UV circular dichroism, intrinsic fluorescence, and thermal melt experiments. We used *E. coli* CysRS as a control. We also investigate the different effects of group I- and some group II-containing salts on the salt-dependent structure of NRC-1 CysRS. Our data shows that this halophilic protein displays salt dependent folding and stabilization of its structure, even in concentrations of salt below NRC-1's intracellular conditions. The protein structure was also observed to be stabilized more effectively by potassium than by other group I metals. However, all group I metals had similar stabilizing effects which indicate that group I metals, in general, can improve halophilic protein stability. Group II metals were significantly less stabilizing towards the overall structure of NRC-1 CysRS. In contrast, the non-halophilic CysRS, *E. coli* CysRS, showed destabilization of its structure as the concentration of salt exceeded 500 mM KCl. These data show that the NRC-1 CysRS has prominent halophilic adaptations that interact best with group I metals, especially potassium. Our conclusions should help improve our overall understanding of protein stability and help to engineer proteins that are particularly salt stable for biotechnology work.

## Materials and Methods

### General

The salts used (LiCl, KCl, NaCl, ZnCl_2_, CsCl, MgCl_2_, CaCl_2_, NiCl_2_, and CuCl_2_) along with solid Tris(hydroxymethyl)aminomethane (Tris) base, sodium phosphate (monobasic and dibasic) and 2-mercaptoethanol (β-Me) were purchased from ThermoFisher Inc. Solid RbCl and imidazole were purchased from Acros Organics. All salt stock solutions (3–3.8M) were made in 50 mM Tris pH 7.5 buffer and were filtered using 0.22 µm Millipore filters. Centrifugation was done using a Beckman Model J2-21 Centrifuge and a Beckman JA-20 rotor. Chromatography was performed on a GE-Healthcare Äkta-Purifier and monitored by inline UV absorbance at 280 nm.

### Expression and purification of NRC-1 CysRS and *E. coli* CysRS

NRC-1 CysRS was expressed heterologously and purified from *Escherichia coli* as described previously [22]. Briefly, cells were lysed using a ThermoIEC French press at 20,000 psi. The pellet was resuspended in 50 mM Tris pH 7.5, 50 mM KCl, and 0.5 mM β-Me and washed with 0.5% deoxycholate. The remaining pellet was resuspended in 8 M urea with 50 mM HEPES pH 7.2, 50 mM KCl and 0.5 mM β-Me. The urea protein solution was dialyzed overnight in a 7.4 pH NaPO_4_ buffer with 0.5 M NaCl, 20 mM imidazole and 0.5 mM β-Me. The protein solution was loaded onto a HisTrap HP nickel-affinity column using a 20 mM sodium phosphate buffer, pH 7.4, with 0.5 M NaCl, 20 mM imidazole and 0.5 mM β-Me. The protein was eluted with a 7.4 pH phosphate buffer with 0.5 M NaCl, 0.5 M imidazole, and 0.5 mM β-Me. For all of the experiments performed, the purified NRC-1 CysRS was dialyzed overnight against a 50 mM Tris pH 7.5 buffer with 10 mM EDTA to remove salts,then dialyzed against 50 mM Tris pH 7.5 overnight to remove EDTA.

*E. coli* CysRS was expressed in *E. coli* and purified as previously described [28]. Purified *E. coli* CysRS was dialyzed into the same solutions as NRC-1 CysRS before CD, fluorescence, and thermal denaturation experiments.

### Fluorescence spectroscopy

Fluorescence and circular dichroism spectroscopy was performed on a JASCO J-815 CD spectrometer with fluorescence attachment and Peltier-type temperature control system. Intrinsic fluorescence emission spectra were collected at room temperature in a 1 cm path length quartz cuvette. The excitation wavelength was 280 nm with a 1 nm bandwidth. The emission spectra were collected from 300 to 400 nm with a bandwidth of 10 nm. The signal was averaged over 10 scans. Protein was diluted to 1 µM in 50 mM Tris pH 7.5 and 0.5 mM β-Me with different concentrations of potassium chloride made from a 3.8 M stock solution. Fluorescence experiments were done independently three times.

### Circular dichroism spectroscopy

Circular dichroism spectra were collected from 300 to 200 nm at room temperature in a 1 cm quartz cuvette. A scanning speed of 50 nm/min was used with a 1 second averaging time and a 0.1 nm step. The signal was averaged over 10 scans. Each spectra was acquired independently three times. Protein was diluted to 1 µM in 50 mM Tris pH 7.5 and 0.5 mM β-Me with various salt solutions. Thermal denaturation experiments were conducted using 5 µM protein in a 1 mm quartz cuvette with various concentrations of potassium chloride and 5% (w/w) glycerol, which was included to prevent evaporation. The samples were heated from 20 to 90°C at a rate of 1.0°C/min, with 3 averaged CD spectra collected from 300 to 200 nm every 2°C (5 nm bandwidth and 100 nm/min scanning speed). Melting transition temperatures (T_M_) and the fractional change in ellipticity were calculated according to Golynskiy, et al. [29], [30]. The T_M_ was calculated by solving for the temperature at which ΔG = 0 in equation 1 using Mathematica, version 9:
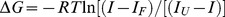
(1)where *I* is ellipticity at 222 nm at temperature *T*, *I_F_* is the folded ellipticity at 4°C, and *I_U_* is the final unfolded ellipticity. The fractional change ellipticity (FCE) was calculated based on equation 2:
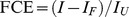
(2)


## Results

### Different salts have a variety of effects on NRC-1 CysRS

It has been shown in the literature that salt induces structural changes in halophilic proteins [19]–[21]. Because it is not established which salt is the most influential on the structure of halophilic proteins, using CD measurements at 222 nm, we surveyed group I, II, and a few transition metals to find the cation that most efficiently stabilizes the structure of NRC-1 CysRS. CD spectroscopy allowed us to detect the presence of secondary structure due to the selective absorbance of one component of circularly polarized light by secondary structural elements, including α-helices, β-sheets, and random coils. These all absorb at specific wavelengths across the UV spectrum. 222 nm was the chosen wavelength since it the major absorption peak for the α-helix. This is a good measure of the CysRS structure, as it is primarily alpha-helical [31], [32].

**Figure 1:**
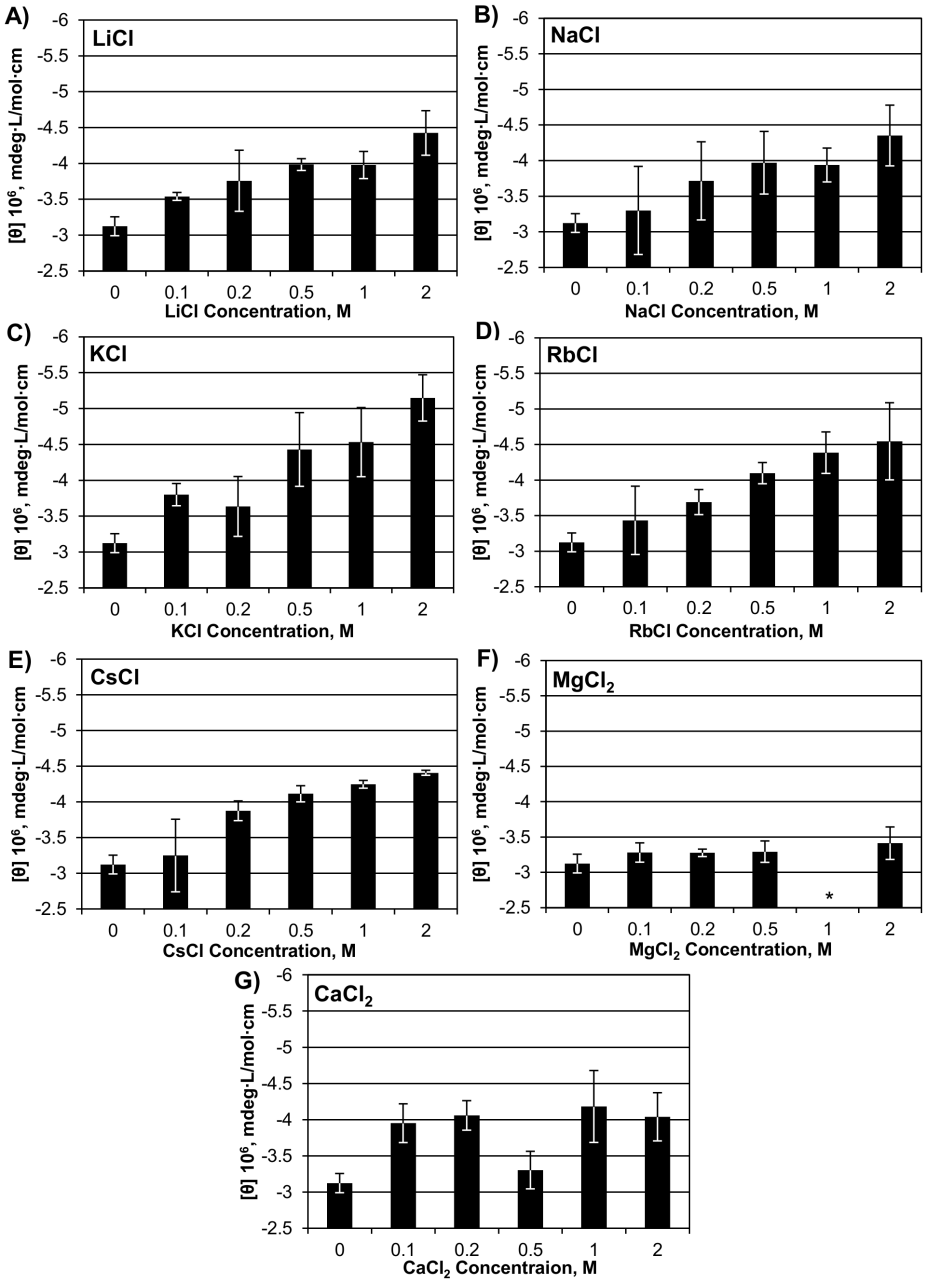
Changes in the ellipticity of NRC-1 CysRS caused by various salts. The circular dichroism signal of NRC-1 CysRS was measured at 222 nm in different concentrations of group I and group II salts: LiCl (A), NaCl (B), KCl (C), RbCl (D), CsCl (E), MgCl_2_ (F), and CaCl_2_ (G). Three independent spectra were recorded and the standard deviation for each concentration is reported as error bars. * A consistent signal for 1 M MgCl_2_ could not be obtained.

CD of NRC-1 CysRS was examined over a range of 0–2 M for the following salts: LiCl, NaCl, KCl, RbCl, CsCl, MgCl_2_, CaCl_2_, NiCl_2_, CuCl_2_, and ZnCl_2_. Chloride salts were used because of their general solubility and to examine the cation-specific effects of each salt without the added effects of changing the counter ion. The concentration limit for this experiment was 2 M, due to solubility limits of some of the stock solutions and, based on our data with KCl, showing that most structural changes were obvious by this concentration [6]. Most group I metals had a similar effect on NRC-1 CysRS (Figure 1), with an average change ranging from 1.5×10^6^ to 2.0×10^6^ millidegrees. Potassium was observed to induce the greatest change in NRC-1 CysRS structure (65% increase in signal) which fits with its potassium rich cellular environment [25]–[27]. Of the divalent cations tested, MgCl_2_ and CaCl_2_ had only a fraction of the effect of group I salts, averaging 0.6×10^6^ millidegrees, suggesting a relatively weak amount of salt-induced folding. While strontium and barium were not tested due to solubility issues, we would expect the same trend as seen with magnesium and calcium. The divalent transition metals, Zn^+2^, Ni^+2^ and Cu^+2^ were also tried; however, interference from NiCl_2_ and CuCl_2_ in the CD signal made testing their effect on NRC-1 CysRS structure not possible (data not shown). Zn^2+^ is an important metal for the active site of CysRS, where it is involved in cysteine discrimination [33]. However, at molar concentrations of ZnCl_2_, there was a denaturing effect on NRC-1 CysRS observed in the CD signal, perhaps due to disruption of the structure (Figure S2).

### Potassium causes NRC-1 CysRS to change its structure

After considering the data in Figure 1, we compared the NRC-1 CysRS potassium induced changes with that of its non-halophilic homolog, *E. coli* CysRS. To do this, we measured CD and fluorescence spectra in the presence of increasing concentrations of KCl for both proteins. While CD was used to make generalizations about its secondary structure, intrinsic fluorescence spectroscopy was used to derive information about the internal structure of the molecule, by monitoring the fluorescence of tryptophans within NRC-1 CysRS. The emission spectra of tryptophan residues can change due to alterations in their environment. Typically, tryptophan emission shifts to lower wavelengths as its environment becomes more hydrophobic [34]–[36]. Likewise, the quantum yield of fluorescence—the relative intensity of the emission spectra—can indicate a change in tryptophan environment [37]. These shifts can show general changes in protein structure based on the position of the tryptophans inside the protein. When pooled together, CD and fluorescence data give us a more complete picture of the overall structure of the enzyme.

**Figure 2:**
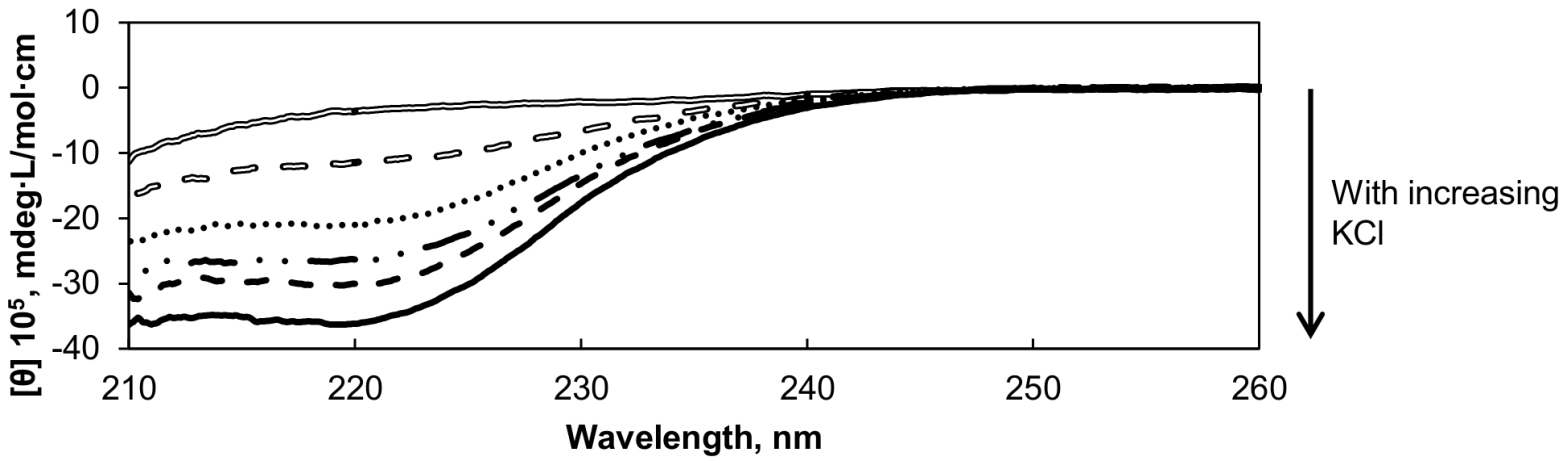
Circular dichroism spectra of NRC-1 CysRS in increasing concentrations of KCl. The CD spectra from 210 to 260-1 CysRS in various concentrations of KCl. As the KCl concentration increases, the CD signal increases. The KCl concentrations are as follows: 50 mM (open line), 100 mM (open dash), 150 mM (dotted line), 200 mM (dash dot line), 1 M (solid dash), 2 M (solid line).

The CD of NRC-1 CysRS in various concentrations of KCl demonstrated that as salt concentration increases, the ellipticity values from 245 nm to 210 nm become more negative (Figure 2). In 2 M KCl, the ellipticity value for NRC-1 CysRS at 222 nm was 10 times lower than the ellipticity of the protein in no salt. This indicated a greater presence of secondary structure which was caused by the increased salt concentration. This salt-induced structural change was reversible; the CD signal decreased after the enzyme was dialyzed against EDTA but it returned when salt was added. There were no changes in the CD signal of the *E. coli* CysRS over the same range of KCl concentrations (Figure S1). This demonstrated that the *E. coli* CysRS structure does not change significantly in different KCl concentrations.

**Figure 3:**
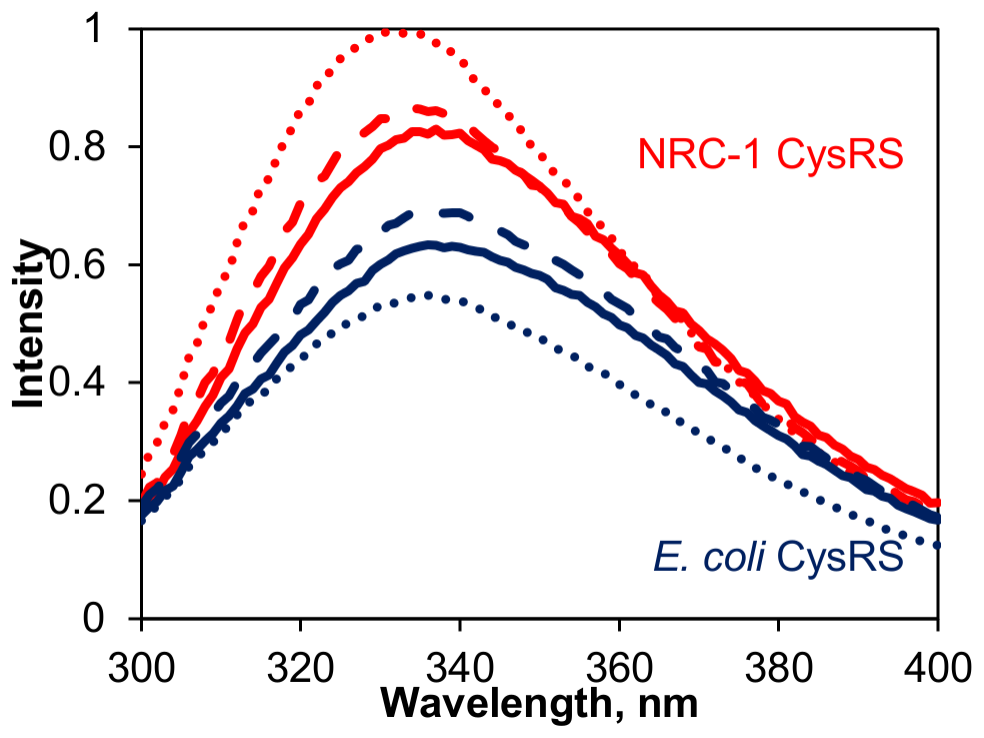
Intrinsic fluorescence emission spectra of NRC-1 (red) and *E. coli (blue)* CysRS. Fluorescence spectra of NRC-1 and *E. coli* CysRS using an excitation wavelength of 280 nm in various concentrations of KCl. The solid line represents the spectra collected in 0 M KCl, the dashed line represents the spectra in 500 mM KCl, and the dotted line represents the spectra collected in 3 M KCl.

The effects of potassium on the structure of NRC-1 CysRS as monitored by intrinsic tryptophan fluorescence had similar results. NRC-1 CysRS contains 8 tryptophan residues in its sequence and their fluorescence spectra are sensitive to their environment [6]. In 0 M KCl, the emission spectrum for NRC-1 CysRS had a peak at 335 nm (Figure 3). As the concentration of potassium increased, this peak shifted to a lower wavelength and the relative quantum yield of fluorescence increased. In 500 mM KCl, the emission maximum shifted to 334 nm with a 5% increase in quantum yield, while at 3 M KCl, the quantum yield of tryptophan fluorescence was 20% greater than 0 M and the emission peak further blue-shifted to 330 nm. The decrease in emission wavelength is indicative of decreased solvent exposure of the tryptophan residues, which could be caused by the burying of the tryptophan residues into the hydrophobic core of the enzyme. The increase in quantum yield confirms that some type of structural change is taking place, since an increased quantum yield means tryptophan is interacting with a different electron acceptor [37]. The observed changes in emission spectra indicate that increasing KCl causes tryptophan residues to become more buried in the protein core, leading to a distinct structural change in NRC-1 CysRS.

For comparison, the tryptophan fluorescence emission spectra of the *E. coli* CysRS were collected in increasing KCl. *E. coli* CysRS only has one less tryptophan than NRC-1 CysRS and, similarly to NRC-1 CysRS, the peak emission wavelength was 336 nm in 0 M KCl. This wavelength did not change in 500 mM or 3 M KCl (Figure 3). The relative quantum yield did change, but in a different way than NRC-1 CysRS. In 500 mM KCl, the quantum yield increased by 9%, but, in 3 M KCl, the quantum yield decreased by 16% compared to *E. coli* CysRS in 0 M KCl. Overall, the *E. coli* enzyme displays different changes in its emission spectra when potassium is increased.

### Potassium increases the stability of NRC-1 CysRS

**Figure 4:**
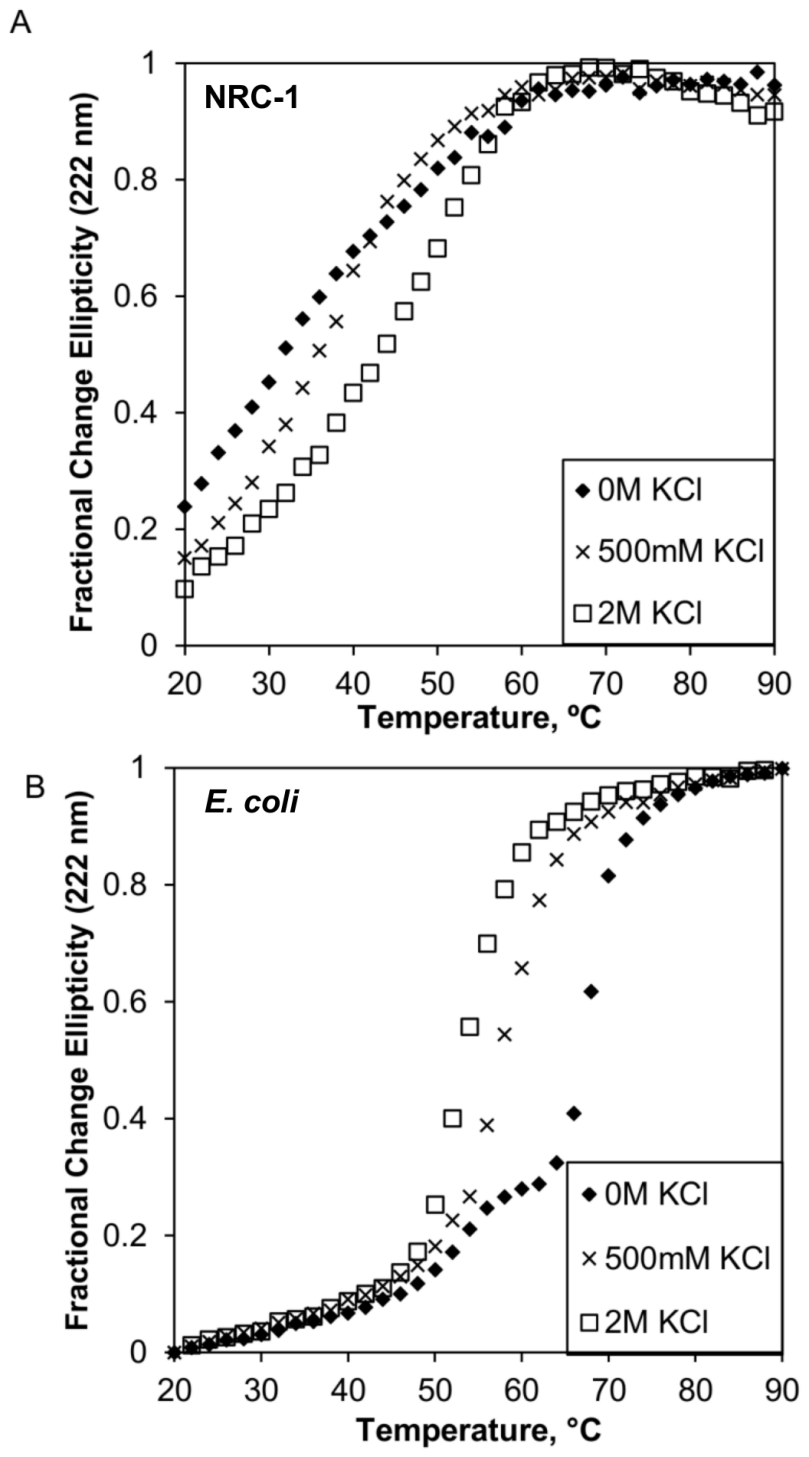
Thermal denaturation curves of NRC-1 and *E. coli* CysRS monitored by CD spectroscopy at 222 nm. Thermal denaturation experiments performed on NRC-1 (A) and *E. coli* (B) CysRS in three concentrations of KCl: 0 M (♦), 500 mM (×), 2 M (□). The midpoint of the curve was used to calculate the melting transition temperatures (TM) for each protein.

The effect of salt on the stability of NRC-1 CysRS was examined by thermal denaturation studies of the enzyme while following the CD signal at 222 nm in 0 M, 500 mM and 2 M KCl. The stability of a protein is defined as the Gibbs free-energy change upon unfolding [12]. However, because thermal denaturation was irreversible for both proteins, ΔG values could not be obtained. Therefore, we used thermal denaturation and T_M_ calculations to compare the relative stability of CysRS. T_M_, or the melting temperature, is the temperature where half of the protein becomes denatured and is an estimate of protein stability. NRC-1 CysRS was observed to unfold with a single transition over the temperature range of 20°C to 90°C (Figure 4A). The transition was irreversible: the CD signal would not decrease after the sample was heated (data not shown). The T_M_ for NRC-1 CysRS in 0 M KCl was 32.1°C (±2.2°C) (Table 1). At 500 mM KCl, the T_M_ appeared to increase slightly to 35.2°C (±0.7°C). A significant increase of the T_M_ was observed when NRC-1 CysRS was heated in 2 M KCl to 42.8°C (±0.5°C), indicating a stabilizing effect by molar concentrations of KCl.

**Table 1:** Melting Transition Temperatures (T_M_) for NRC-1 and *E. coli* CysRS†.

KCl	NRC-1 CysRS (°C)	E. coli CysRS (°C)
0 M	31.1 (±2.2)	66.8 (±0.3)
500 mM	35.2 (±0.7)	57.6 (±0.6)
2 M	42.8 (±0.5)	53.3 (±1.2)

† T_M_ was calculated from CD thermal denaturation curves at 222 nm.

The same experiment was done on CysRS from E. coli, which showed an opposite trend to NRC-1 CysRS (Figure 4B). Despite having little effect on the secondary structure of *E. coli* CysRS, salt decreased the enzyme's stability. Without salt, E. coli CysRS was observed to melt with two transitions, having a calculated melting temperature at the higher transition of 66.8°C (±0.3°C) (Table 1), and precipitation was observed at the end of each melt. In 500 mM and 2M KCl, the two transitions became a single one and the melting temperature significantly decreased to 57.6°C (±0.6°C) and 53.3°C (±1.2°C), respectively.

## Discussion

Salt has drastically different effects on cysteinyl-tRNA synthetases from the halophilic *H. salinarum* spp. NRC-1 and the mesophilic *E. coli*. The large change in fluorescence emission spectra of NRC-1 CysRS in high salt compared to the *E. coli* version suggests that the halophilic version is more structurally sensitive to its ionic environment (Figure 3). This was supported by CD spectra of NRC-1 CysRS, which demonstrated large changes in the ellipticity of the CD spectra with increasing potassium, indicative of significant structural changes within the enzyme (Figure 2). The changes in CD spectra were reversible: when salt was added to the NRC-1 CysRS its ellipticity decreasd, but diluting the protein from a high salt buffer to low salt (data not shown) saw an increase in ellipticity. These results support the idea that this synthetase folds in the presence of salt, independent from the cell. This suggests that it is not folded via a chaperonin-dependent manner. The *E. coli* enzyme did not show the same structural effects in salt, demonstrating the ability of *H. salinarum* ssp. NRC-1 to utilize its extreme environment for protein folding. These results support previous findings that halophilic proteins gain a significant amount of their protein structure by being in a high salt environment [19]–[21]. Furthermore, our spectra of the NRC-1 CysRS in this work match past spectra taken of the enzymatically active protein [6]. For this reason, it is possible that the loss of activity in the NRC-1 CysRS below 2 M KCl is not due a structural effect. Likewise, our spectra of the *E. coli* CysRS show that its loss of activity in salt is also not due to a structural effect. While the reason for the loss in activity of the *E. coli* CysRS in high salt is unknown, previous works suggests that salt interferes with the active site chemistry [6].

The salt-dependent structure of NRC-1 CysRS also appeared to be affected by the type of salt (Figure 1). K^+^ was the strongest in inducing secondary structure in NRC-1 CysRS while other group I ions had a similar, but slightly reduced effect. The divalent group II ions had only a small effect on NRC-1 CysRS structure. These results may be a consequence of the stabilizing effect, or lack thereof, of certain cations on the native fold of proteins. Metals cations have the ability to influence protein stability through interacting with water and controlling the hydration of the protein. The Hofmeister series predicts that the most structure-promoting cations are ones that have low charge density and weakly bind water [38]. As the group I metal ions increase in size, their positive charge is more widely dispersed across the ion, giving them weaker interactions with a greater number of water molecules. Therefore, larger group I cations would be expected to have a more stabilizing effect on NRC-1 CysRS. Along these lines, the strong effects of Cs^+^, Rb^+^, and Na^+^ on the structure of NRC-1 CysRS are to be expected, because these metals promote structure in the protein. The group II divalent cations typically have stronger interactions with water due to the greater charge. The weaker effects of Mg^2+^ and Ca^2+^ observed could therefore be explained by the Hofmeister effect. What is not expected by the Hofmeister series, though, was K^+^ having the strongest effect on NRC-1 CysRS structure, since it is a more chaotropic salt (structure destabilizing) than Na^+^, Rb^+^, and Cs^+^. The high effect of K^+^, then, may be due to evolutionary selection, since it is the most abundant cation in the intracellular environment of *H. salinarum* ssp. NRC-1 (4.7 M) [25], [26]. Studies of the Hofmeister effect on a halophilic malate dehydrogenase, found a similar order of stabilizing cations as the ones reported here [38]. Ebel et al. concluded that the stabilization of a halophilic protein caused by a cation with high charge density could indicate specific protein-salt interactions that are present in the folded protein. Not observed in the halophilic malate dehydrogenase was the increased effect of potassium ions. This effect in NRC-1 CysRS, though, supports the hypothesis of specific salt binding, which could select for potassium, an advantage for the protein since it is so abundant in the cytoplasm. Protein binding optimized for potassium would have to involve more than just the carboxylate groups of the acidic residues on the surface of NRC-1 CysRS. Carboxylate groups, by themselves, have a greater affinity for sodium ions than potassium [39]. Therefore, the higher affinity for potassium may be a consequence of the arrangement of acidic residues along with other polar groups, which could include carbonyl groups from the backbone or polar side groups.

This work has shown that, like many other extremophiles, halophiles are capable of using their environment to help fold their proteins and keep their folded form stable. Here, it appears that salt-protein interactions replace the usual electrostatic interactions that proteins use to remain folded correctly. Support for this is found in the thermal denaturation experiments conducted on *E. coli* and NRC-1 CysRS. In the E. coli version of the enzyme, the thermal denaturation shifted from a double-transition curve in no salt to a single transition in 500 mM KCl. The lost transition could correlate to the disruption of stabilizing electrostatic interactions which are not able to form in highly ionic environments. This is a compelling explanation for *E. coli* CysRS's reduced stability in high salt. In contrast, NRC-1 CysRS becomes more stable in high salt, suggesting external, stabilizing salt-protein interactions instead of intramolecular electrostatic interactions. This salt-protein interaction would most likely be due to favorable interactions between the negatively charged acidic surface residues on the halophilic protein and the positively charged metal ion of the salt.

Another observation from the thermal denaturation experiments was that the melting temperatures of *E. coli* CysRS were consistently higher than NRC-1 CysRS for all the salt conditions tested. It is unclear whether this is an inherent difference between the two enzymes or if it is a consequence of non-physiological experiment conditions. The stability of NRC-1 CysRS would be expected to increase further past 2 M KCl, especially since the intracellular potassium concentration of *H. salinarum ssp.* NRC-1 is near 4 M [27]. It is also possible that reduced thermal stability is due to the common halophilic adaptation of a smaller hydrophobic core [17]. A smaller hydrophobic core would make NRC-1 CysRS consistently less resistant to thermal denaturation than its mesophilic counterpart. Improving the strength of the hydrophobic core by increasing the ionic strength of the solvent may be another cause of improved thermal stability in the halophilic CysRS.

Overall, our findings on the salt-dependent structure and stability of NRC-1 CysRS support previous research that showed the same effects in different halophilic proteins. This demonstrates how halophilic proteins utilize their salty environment to fold into a functional form by stabilizing protein structure in high salt concentrations. Also, NRC-1 CysRS's apparent specificity for potassium ions suggests that evolution has selected for strong protein-salt interactions with that cation, a consequence, most likely, from the prevalence of that ion in the intracellular environment of *H. salinarum* NRC-1.

## Supporting Information

Figure S1 **CD signal of NRC-1 CysRS in ZnCl_2_ at 222 nm.** CD ellipticity of 1 µM NRC-1 CysRS in various concentrations of ZnCl_2_ at 222 nm. The standard deviation of three separate spectra is shown as error bars.
(TIF)

Figure S2 **CD signal of** ***E. coli*** **CysRS in KCl at 222 nm.** CD ellipticity of 1 µM *E. coli* CysRS in various concentrations of KCl at 222 nm. The standard deviation of three separate spectra is shown as error bars.
(TIF)
