# Supplementary figures and images for: Circular Dichroism and Fluorescence Spectroscopy of Cysteinyl-tRNA Synthetase from Halobacterium salinarum ssp. NRC-1 Demonstrates that Group I Cations Are Particularly Effective in Providing Structure and Stability to This Halophilic Protein
Source: PLoS One. 2014 Mar 3;9(3):e89452. doi: 10.1371/journal.pone.0089452 (PMC3940603; doi:10.1371/journal.pone.0089452)

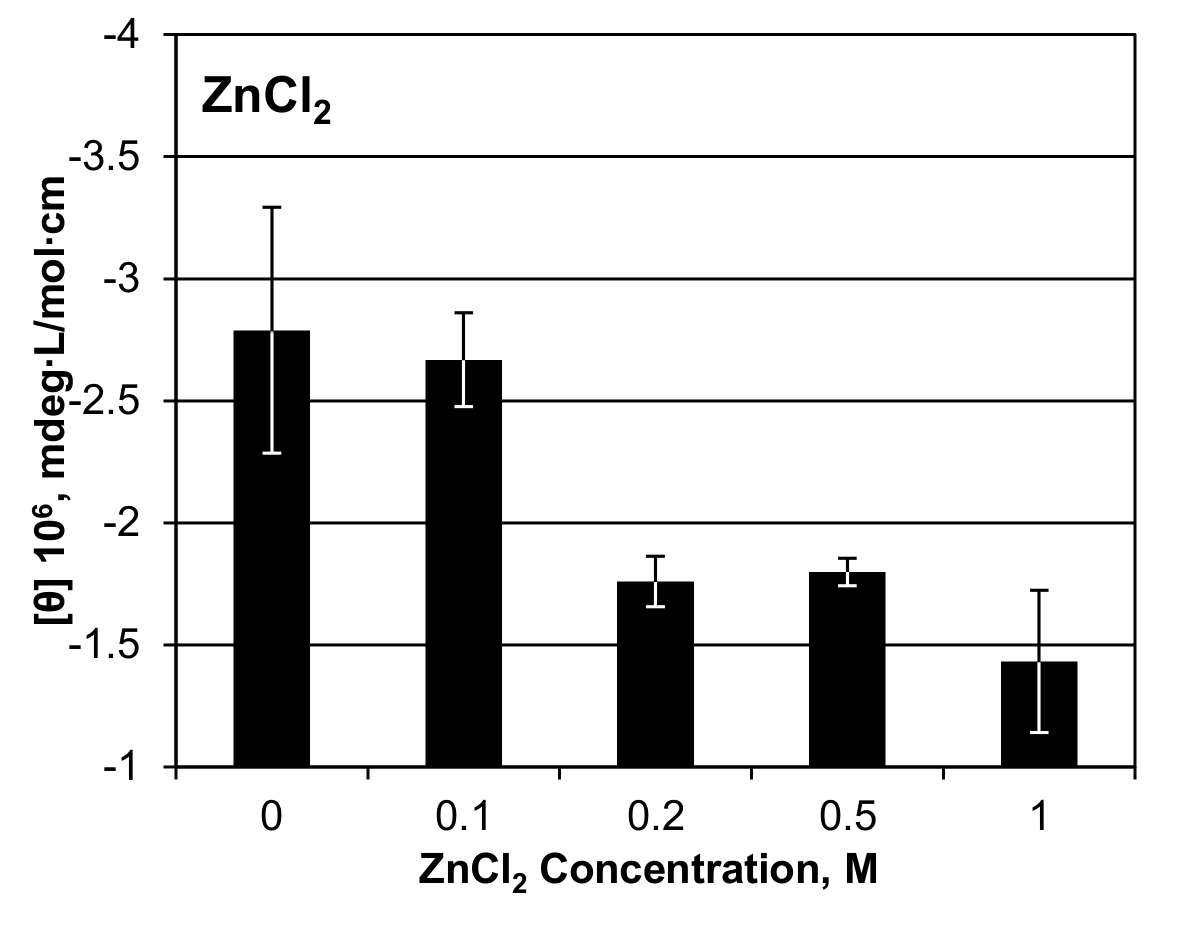

Supplement: Figure S1 — CD signal of NRC-1 CysRS in ZnCl2 at 222 nm. CD ellipticity of 1 µM NRC-1 CysRS in various concentrations of ZnCl2 at 222 nm. The standard deviation of three separate spectra is shown as error bars. (TIF) [file pone.0089452.s001.tif]

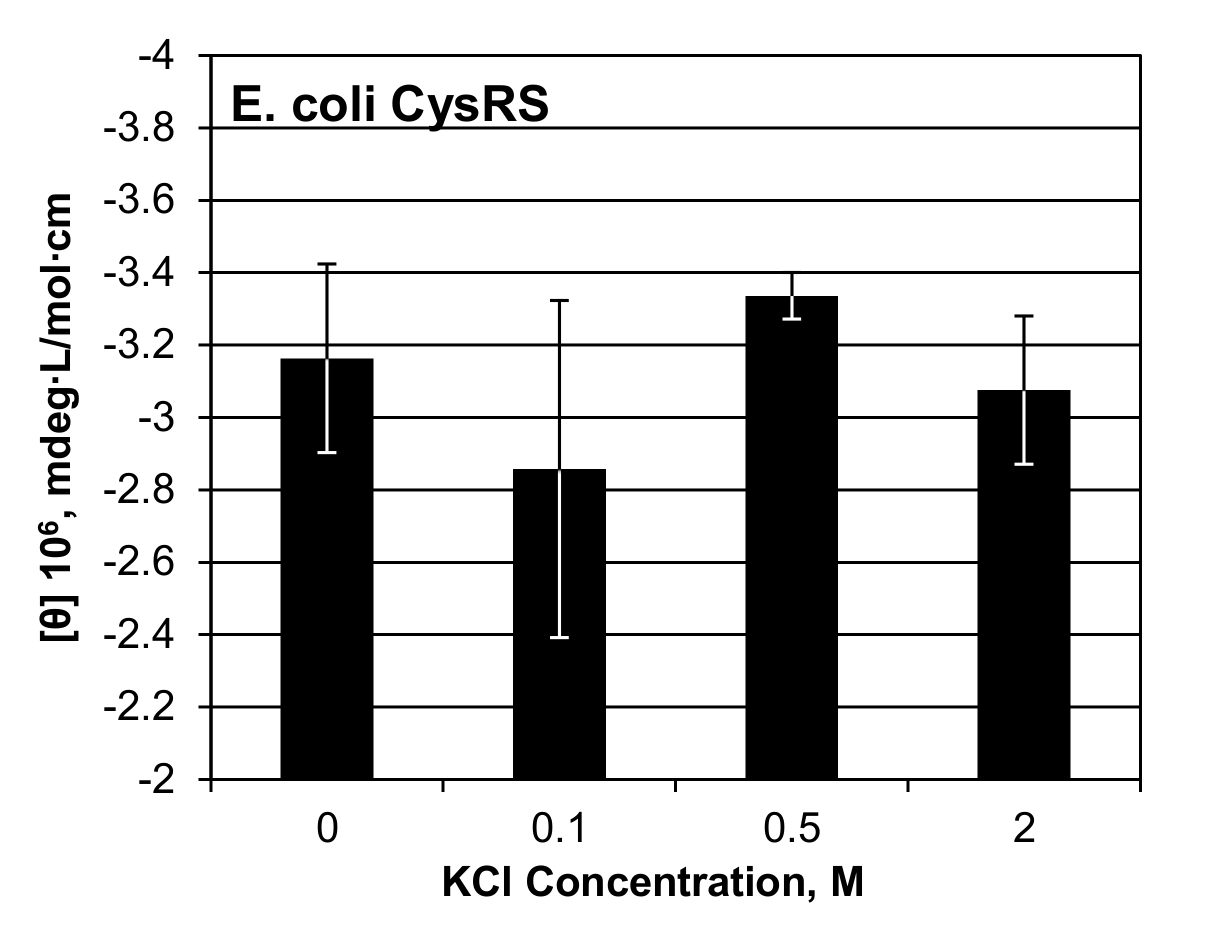

Supplement: Figure S2 — CD signal of E. coli CysRS in KCl at 222 nm. CD ellipticity of 1 µM E. coli CysRS in various concentrations of KCl at 222 nm. The standard deviation of three separate spectra is shown as error bars. (TIF) [file pone.0089452.s002.tif]
